# Quantifying nanoparticles in clays and soils with a small-angle X-ray scattering method

**DOI:** 10.1107/S1600576719017266

**Published:** 2020-02-01

**Authors:** Katsuhiro Tsukimura, Masaya Suzuki

**Affiliations:** a Research Institute of Geo-resources and Environment, Geological Survey of Japan, Advanced Industrial Science and Technology, Central 7, Higashi-1, Tsukuba, Ibaraki 305-8567, Japan

**Keywords:** small-angle X-ray scattering, nanoparticles in clays and soils, quantitative analysis of nanoparticles, colloidal silica, silica gel, kaolinite, smectite, pyrophillite, allophane, ferrihydrite

## Abstract

Clays and soils produce strong small-angle X-ray scattering (SAXS) because they contain large numbers of nanoparticles, namely allophane and ferrihydrite. These nanoparticles are amorphous and have approximately spherical shape with a size of around 3–10 nm. The weight ratios of these nanoparticles will affect the properties of the clays and soils. However, the nanoparticles in clays and soils are not generally quantified and are sometimes ignored because there is no standard method to quantify them. This paper describes a method to quantify nanoparticles in clays and soils with SAXS. This is achieved by deriving normalized SAXS intensities from unit weight of the sample, which are not affected by absorption. By integrating the normalized SAXS intensities over the reciprocal space, one obtains a value that is proportional to the weight ratio of the nanoparticles, proportional to the square of the difference of density between the nanoparticles and the liquid surrounding the nanoparticles, and inversely proportional to the density of the nanoparticles. If the density of the nanoparticles is known, the weight ratio of the nanoparticles can be calculated from the SAXS intensities. The density of nanoparticles was estimated from the chemical composition of the sample. Nanoparticles in colloidal silica, silica gels, mixtures of silica gel and α-aluminium oxide, and synthetic clays have been quantified with the integral SAXS method. The results show that the errors of the weight ratios of nanoparticles are around 25% of the weight ratio. It is also shown that some natural clays contain large fractions of nanoparticles; montmorillonite clay from the Mikawa deposit, pyrophillite clay from the Shokozan deposit and kaolinite clay from the Kanpaku deposit contain 25 (7), 10 (2) and 19 (5) wt% nanoparticles, respectively, where errors are shown in parentheses.

## Abstract 2

This paper describes a method to quantify nanoparticles in clays and soils with small-angle X-ray scattering.

## 1. Introduction   

Clays and soils produce strong small-angle X-ray scattering (SAXS) (*e.g.* Miyoshi *et al.*, 2015 ▸), which implies that they contain large numbers of nanoparticles. These nanoparticles will be allophane (Wada & Wada, 1976 ▸; Theng & Yuan, 2008 ▸; Calabi-Floody *et al.*, 2011 ▸) or ferrihydrite (Yuwono *et al.*, 2012 ▸), which are amorphous and have a shape close to spherical with a size of around 3–10 nm. Quantifying nanoparticles in clays and soils is important because the weight ratios of these nanoparticles will affect the properties of the clays and soils, such as cation exchange capacity, gelation properties, plasticity and water permeability. However, nanoparticles in clays and soils are not generally quantified and are sometimes ignored because there is no standard method to quantify them. Therefore, we have devised a method to quantify nanoparticles in clays and soils.

We use an integral SAXS method. By integrating the SAXS intensities over the reciprocal space, we can obtain a value that is proportional to the weight ratio of the nanoparticles. Toraya (2016 ▸) applied this integral method to quantify crystals. We adopt this method because it is simple and easy: we do not need to calculate the SAXS intensities from the size distribution and aggregation state of the nanoparticles. Although this integral SAXS method is easy to perform, we still have several problems.

The first problem is that raw SAXS intensities are affected by mass and absorption effects. The raw SAXS intensities are proportional to the mass of the sample, and the SAXS intensities decrease with increasing absorption effects. We have found that these effects can be corrected from the intensity of the incident beam, the transmission factor and the chemical composition of the sample. With this method, we have transformed raw observed intensities to normalized observed intensities, where the normalized observed intensities are the intensities scattered by unit weight of the sample with no absorption effect. By integrating the normalized intensities, we have obtained the integral intensity of the SAXS, which is proportional to the weight ratio of nanoparticles.

The second problem is that we have to know the density of the nanoparticles because the integral intensity of SAXS is proportional to the weight ratio of nanoparticles, proportional to the square of the difference of density between the nanoparticles and the liquid surrounding the nanoparticles, and inversely proportional to the density of the nanoparticles. Therefore, we need to determine the density of nanoparticles to calculate the weight ratio of nanoparticles from the integral intensity of SAXS. However, measuring the density of nanoparticles in clays and soils is difficult. Therefore, we have estimated the density of nanoparticles from the chemical composition of the sample. In this estimation, we assume that the chemical composition of the nanoparticles is the same as that of the whole sample, and also assume that the volume of nanoparticles is proportional to the weight ratio of oxygen atoms in the nanoparticles.

The third problem is that the scale factor which is needed for determining the weight ratio of nanoparticles from the integral SAXS intensity is unknown. To determine the scale factor, we have measured a standard sample whose weight ratio of nanoparticles is known. We used colloidal silica as the standard sample because it is commercially available and contains a large, known quantity of nanoparticles. Because the scale factor depends on the optical system of the diffractometer, we need to redetermine the scale factor when we use a new diffractometer or when we change the optical system of the diffractometer.

The fourth problem is that the SAXS signals from nanoparticles are sometimes overlapped with a diffraction peak from a clay mineral. In particular, the strong broad 001 peak of smectite, which is present between 3 and 8° 2θ (Cu * K*α), sometimes overlaps with the SAXS from nanoparticles. In that case, we have to remove the peak of the clay mineral from the SAXS. Such peaks can be fitted with a normal distribution function or the summation of two normal distribution functions.

The fifth problem is that it is unclear whether or not SAXS of clays or soils comes only from nanoparticles. Therefore, we have fitted the observed SAXS intensities with calculated ones that are derived from the size distribution and aggregation state of the nanoparticles. Using the scale factor between the observed SAXS intensities and the calculated ones, we have also calculated the weight ratio of nanoparticles. Therefore, we can compare this weight ratio with that determined with the integral SAXS method. If these two values are the same, these results support the hypothesis that SAXS of clays and soils comes only from nanoparticles.

We have used our integral SAXS method to quantify nanoparticles in colloidal silica, silica gels, mixtures of silica gel and α-aluminium oxide, synthetic clays, and natural clays. The colloidal silica, silica gels, mixtures of silica gel and α-aluminium oxide, and synthetic clays were measured in order to estimate the errors of the integral SAXS method because they have known fractions of nanoparticles. Natural clays were measured to show the procedure to quantify nanoparticles and to show that natural clays contain large quantities of nanoparticles.

## 2. Theory   

### 2.1. Derivation of normalized scattering intensities   

Table 1 ▸ shows the symbols used in this paper. To quantify the nanoparticles with the integral SAXS method, we have derived normalized SAXS intensities (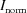) from raw SAXS intensities (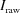), where the normalized SAXS intensities are the intensities scattered by unit weight of a sample from unit intensity of the incident beam with no absorption effect. The value of the raw SAXS intensity is proportional to the intensity of the incident beam (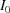), the transmittance factor (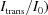, the mass of the sample (*M*) and the normalized SAXS intensity (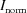):
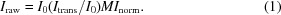
The mass of the sample can be calculated with the equation
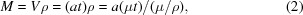
where *V*, ρ, *a*, *t* and μ are the volume, the density, the cross section, the length and the absorption coefficient of the sample. The value of the cross section (*a*) is constant unless the optical system is changed. Because we can include the value of *a* in the *K* value in equation (9) below, we do not need to determine the value of *a*. Therefore, we temporarily set the value of *a* as unity. 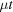 is calculated with the following equation:
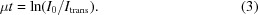


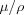
 is calculated with the following equation:
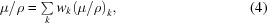
where 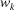 is the weight ratio of element *k* and (μ/ρ)_*k*_ is the mass absorption coefficient of element *k*. Mass absorption coefficients are listed in *International Tables for Crystallography*, Vol. C (2006 ▸).

### 2.2. Relation between the integral SAXS intensity and the weight ratio of nanoparticles   

The SAXS intensity is proportional to the Fourier transform of the Patterson function of nanoparticles:
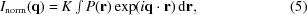
where *K* is a scale factor. The inverse Fourier transform of the scattering intensity gives the Patterson function:
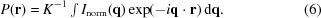
The Patterson function at 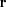 = **0** becomes
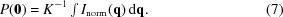
The Patterson function at 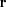 = 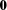 is further expressed as
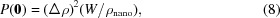
where *W* is the weight ratio of nanoparticles in a sample, 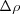 is the difference of density between the nanoparticles and the liquid surrounding the nanoparticles, and 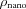 is the density of the nanoparticles. The derivation of equation (8) is shown in Appendix *A* [App appa]. From equations (7) and (8), we can derive the relation
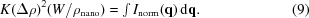
By measuring SAXS intensities of a sample with a known content of nanoparticles (*W*), we can determine the value of *K* from the integral SAXS intensity and the densities of the nanoparticles and the liquid surrounding the nanoparticles. After we determine the value of *K*, we can calculate the weight ratio of nanoparticles in another sample from the integral SAXS intensity and the densities of the nanoparticles and the liquid surrounding the nanoparticles.

### 2.3. Calculation of SAXS intensities on the basis of the size distribution and aggregation state of nanoparticles   

This section shows how we calculate the SAXS intensities of nanoparticles on the basis of their size distribution and aggregation state. We consider a polydisperse system consisting of *L* kinds of nanoparticles with spherical symmetry. We define the function 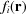 as the density distribution of the *i*th (*i* = 1 to *L*) kind of nanoparticle. We consider the case that the concentration of nanoparticles is so high that the interference between two nanoparticles is significantly large. To describe the interference between two nanoparticles, we introduce a function 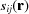, which shows the probability that we find the *j*th kind of nanoparticle at the position 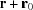 when the *i*th kind of nanoparticle is at the position 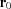. We also assume that 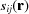 has spherical symmetry. Using 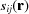, we can express the Patterson function 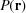 as
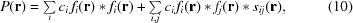
where 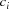 is 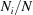 (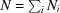; 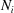 is the number of nanoparticles of the *i*th kind) and the symbol ‘
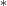
’ denotes the convolution. The second term is called the interference term. We approximate 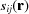 as
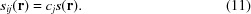
This approximation is called the decoupling approximation (Kotlarchyk & Chen, 1983 ▸; Stieger *et al.*, 2004 ▸; Eyssautier *et al.*, 2011 ▸), where 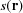 is the probability that we find the center of one nanoparticle at the position 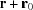 when the center of another nanoparticle is at the position 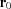. Note that the function 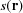 is independent of the kind of nanoparticle. By substituting approximation (11) into equation (10), we have the approximated Patterson function:
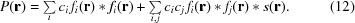
The Fourier transform of 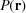 gives the scattering intensities:
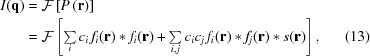
where 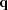 is the position in reciprocal space. Because the Fourier transform is linear and because the Fourier transform of the convolution of two functions is equivalent to the multiplication of the Fourier transforms of these two functions, we can express the scattering intensities as
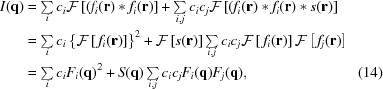
where 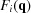 and 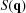 are the Fourier transforms of 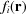 and 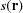, respectively. 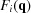 and 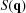 are called the form factor and structure factor, respectively. The derivation of form factors 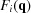 is shown in Appendix *B* [App appb], the derivation of the number ratios of the *i*th nanoparticles (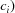 in Appendix *C* [App appc] and the derivation of structure factors 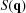 in Appendix *D* [App appd].

## 3. Experimental   

### 3.1. Samples   

The samples that we used in this study were colloidal silica, silica gels, mixtures of silica gel and α-aluminium oxide, synthetic clays, and natural clays. Table 2 ▸ describes these samples, and Tables 3 ▸ and 4 ▸ show their chemical compositions. The amounts of water shown in Tables 3 ▸ and 4 ▸ are the summation of water in solids and in liquid.

The colloidal silica and silica gels were prepared from LUDOX SM30, which was purchased from Grace Davison. LUDOX SM30 is colloidal silica consisting of nanoparticles with a radius of 3.5 nm and contains 30 wt% silica. The samples SM20, SM10, SM5 and SM2 were prepared by diluting SM30 with water, where SM20, SM10, SM5 and SM2 are colloidal silica containing 20, 10, 5 and 2 wt% silica, respectively. The samples SMG71 and SM90, which are silica gel with 71.2 and 89.7 wt% silica, respectively, were prepared by evaporating water from SM30 at room temperature. Although we evaporated as much water as possible for SMG71 and SMG90, the amounts of water evaporated from SM30 differ for SMG71 and SMG90. SMG71 was prepared from the colloidal silica two years after purchase, and SMG90 was prepared immediately after purchase. Because the vis­cosity of the colloidal silica increases with time, the aggregation of nanoparticles will proceed with time. This is the reason for the large amount of water retained in silica gel SMG71.

The synthetic clays contain kaolinite and amorphous nanoparticles and were synthesized hydro­thermally at 473 K for 49 or 280 days from aluminium–silica gels with the Al/Si mole ratio of 3/7, 4/6 and 5/5 in the study of Tsukimura (1997 ▸). Kaolinite ratios in these samples were also taken from Tsukimura (1997 ▸), where the kaolinite ratios were determined from the intensity of the 001 reflection with the internal standard method of Alexander & Klug (1948 ▸). We calculated the fraction of nanoparticles in these samples on the assumption that the samples contain only kaolinite and amorphous nanoparticles.

The three natural clays analyzed in this study were taken from the reference samples of the Clay Science Society of Japan. One is montmorillonite clay from the Mikawa deposit, central Japan, another is pyrophyllite clay from the Syokozan deposit, western Japan, and the other is kaolinite clay from the Kanpaku deposit, central Japan. These natural clays contain clay minerals as a major component: *i.e.* montmorillonite, pyrophillite and kaolinite, respectively. The montmorillonite clay contains small amounts of cristobalite, mica and amphibole, the pyrophillite clay contains a small amount of quartz, and the kaolinite clay contains small amounts of alunite and quartz (Miyawaki *et al.*, 2010 ▸). The chemical compositions of the nanoparticles in these natural clays were not measured, but they will be close to that of the whole sample. This is because most nanoparticles precipitate before a clay mineral crystallizes, and because the chemical composition of the clay mineral is also close to that of the whole sample (Tsukimura, 1997 ▸).

### 3.2. Estimation of density of nanoparticles   

**Figure 1:**
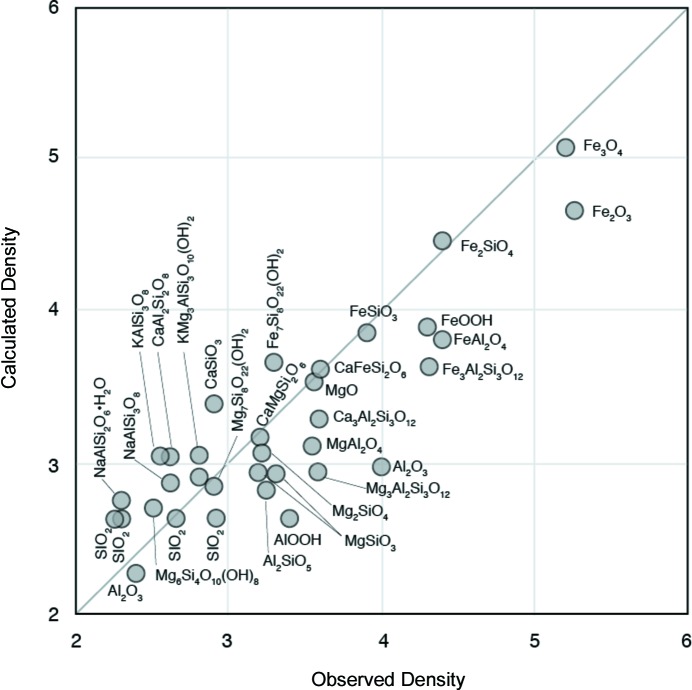
Calculated densities as a function of observed densities for silicate and oxide minerals. When calculating the density, we assumed that the volume of the mineral depended only on the number of oxygen atoms and its volume was 11.41 cm^3^ for one mole of oxygen.

The observed density of nanoparticles in the colloidal silica and silica gels was 2.27 g cm^−3^, which was derived from the density of the colloidal solution SM30. Because the densities of nanoparticles in clays and soils cannot be measured with this method, we have estimated the densities of nanoparticles from the chemical composition of the samples. In this estimation, we assume that the chemical composition of the nanoparticles is as same as that of whole clay sample, and also assume that nanoparticles in the sample consist of oxygen and several kinds of cations. Because the ionic radius of oxygen is large compared with those of the cations, we consider that the space in the minerals and nanoparticles is filled with oxygen atoms and the cations enter into the interstices of the oxygen atoms. Therefore, we assume that the volumes of minerals and nanoparticles are a function of only the number of oxygen atoms and that the volume of oxygen is 11.41 cm^3^ per mole. We also assume that the nanoparticles do not contain water. Fig. 1 ▸ shows the densities of the silicate and oxide minerals estimated with this method as a function of the observed densities. The standard deviation of the difference between the calculated densities and the observed densities is 12% of the density.

### 3.3. Measurement of SAXS intensities   

We measured the SAXS intensities with a multipurpose X-ray diffractometer (Ultima IV, Rigaku), setting the optical system for SAXS measurement. The samples were placed into borosilicate capillaries of 0.5 or 0.7 mm in diameter. The incident beam was Ni-filtered Cu *K*α (λ = 0.154184 nm) radiation from an X-ray tube at 40 kV and 50 mA. The intensities of the incident beam and transmitted beam were measured using an aluminium absorber. The scattering intensities were measured from 0.10 to 12.00° 2θ with a step width of 0.02° and a scan speed of 0.1° min^−1^.

### 3.4. Analysis of small-angle X-ray intensity data   

**Figure 2:**
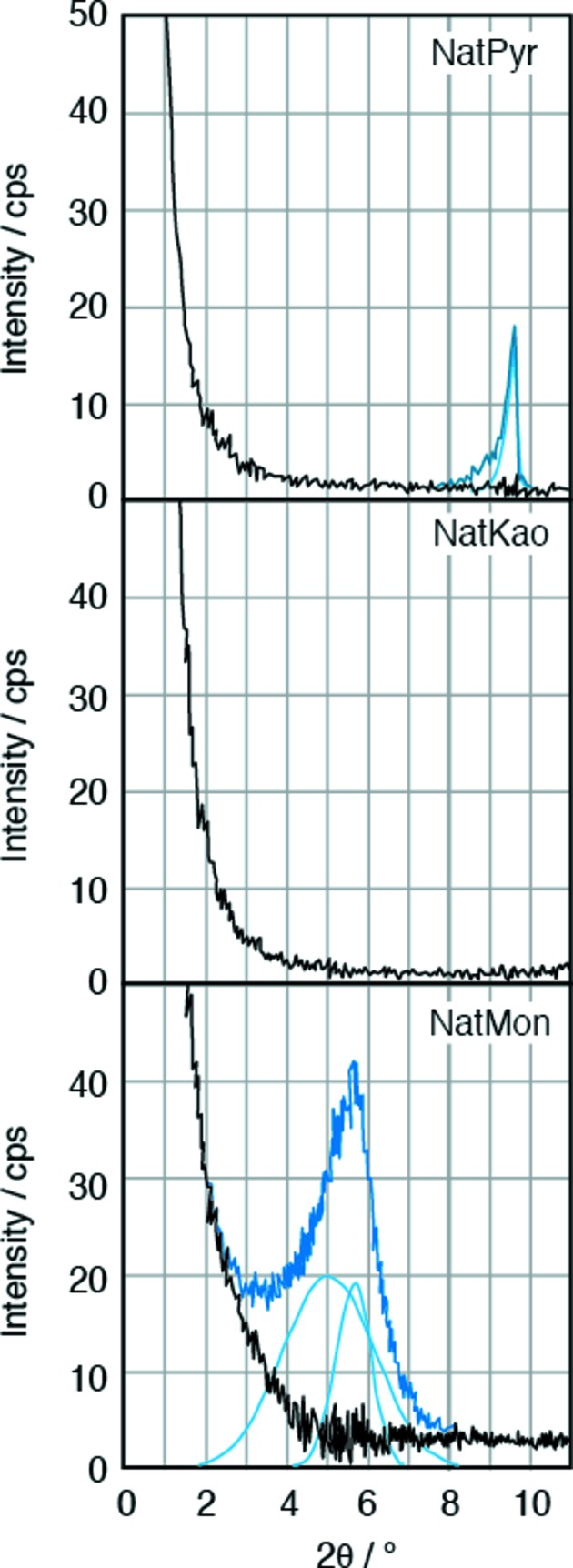
SAXS curves of NatKao, NatPyr and NatMon. When a diffraction peak from a clay mineral was present in the area (2θ < 10°), the peak was removed from the scattering curve on the assumption that the peak is the summation of one or two normal distributions.

**Figure 3:**
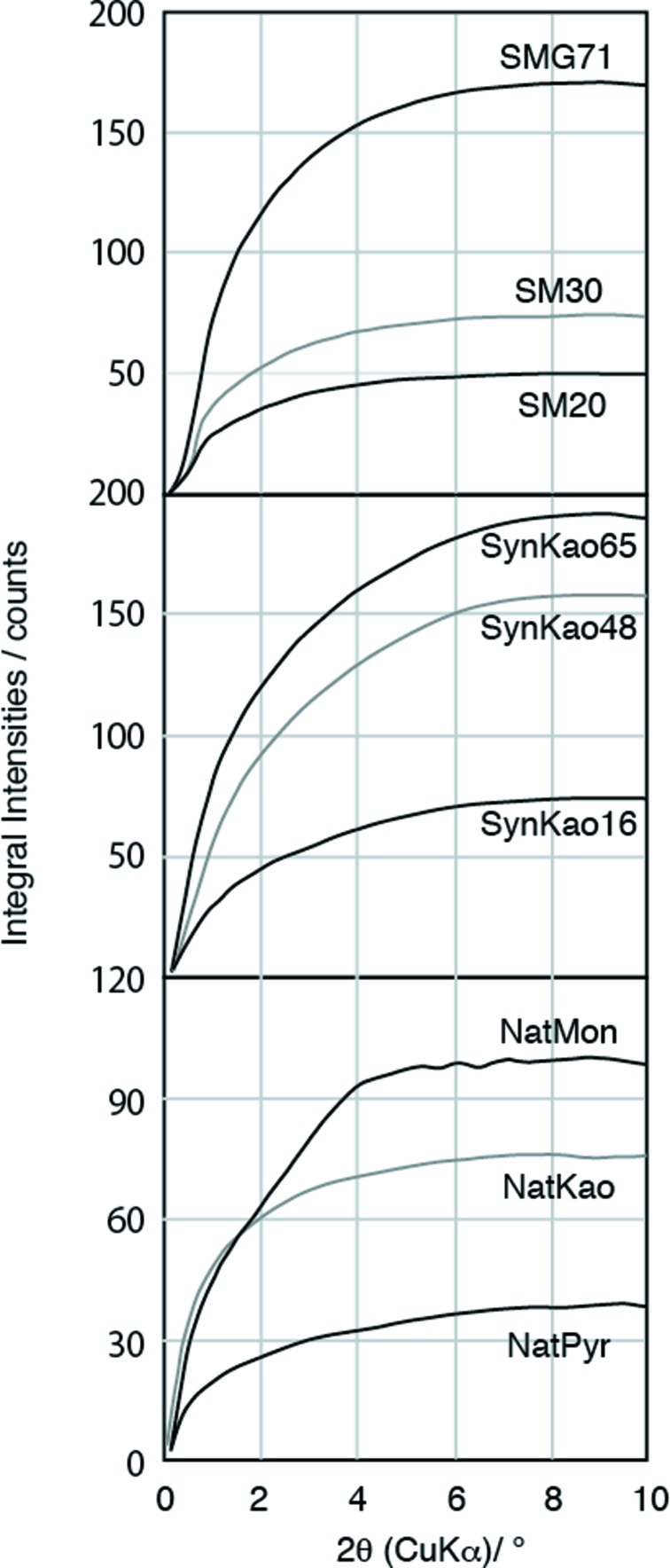
Integrated values of SAXS intensities as a function of the end point of integration. Integrated intensities monotonically increase up to 8° 2θ and become nearly constant above 8°.

We transformed the raw intensities (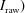 to normalized intensities 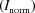 using equation (1). This calculation uses the intensities of the incident direct beam (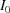) and transmitted direct beam (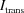) and the chemical composition. When a peak from a clay mineral was present at 2θ less than 10°, the peak was removed from the normalized intensities (Fig. 2 ▸). The peak was approximated with one or two normal distribution functions that were corrected for smearing effects. The background was subtracted from the normalized intensities, where the background was calculated by averaging the intensities around 9° in 2θ. Then, the intensities were integrated in three-dimensional reciprocal space up to 8° in 2θ. We assumed that 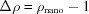, which implies that the nanoparticles are surrounded by water whose density is 1. We calculated the scale factor (*K*) from the integral intensity of SM30 using equation (9). After the scale factor had been determined, we calculated the weight ratio of nanoparticles in the other samples from the integral SAXS intensity using equation (9). Fig. 3 ▸ shows the integral intensities as a function of end point of integration. The integral intensities increase monotonically up to 8° 2θ, and the value becomes constant above 8° 2θ. Therefore, we have finished integration at 8° 2θ.

The size distributions and aggregation states of nanoparticles were estimated using the intensity data up to 3° 2θ. We assumed that the shape of nanoparticles was spherical with a spherical void at the center, and that the radius of the void was the same for all the nanoparticles in a sample. We also assumed that the distribution of the total volume of spheres was expressed with a volume logarithmic normal distribution. We included an interference term in the calculation of SAXS intensities because the interference effect cannot be neglected. The interference effect was calculated using the decoupling approximation method (Kotlarchyk & Chen, 1983 ▸) as shown in equation (11). Parameters were adjusted such that the residual factor had the least value, where the residual factor is
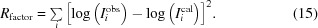



## 4. Results   

### 4.1. Weight ratios of nanoparticles   

**Figure 4:**
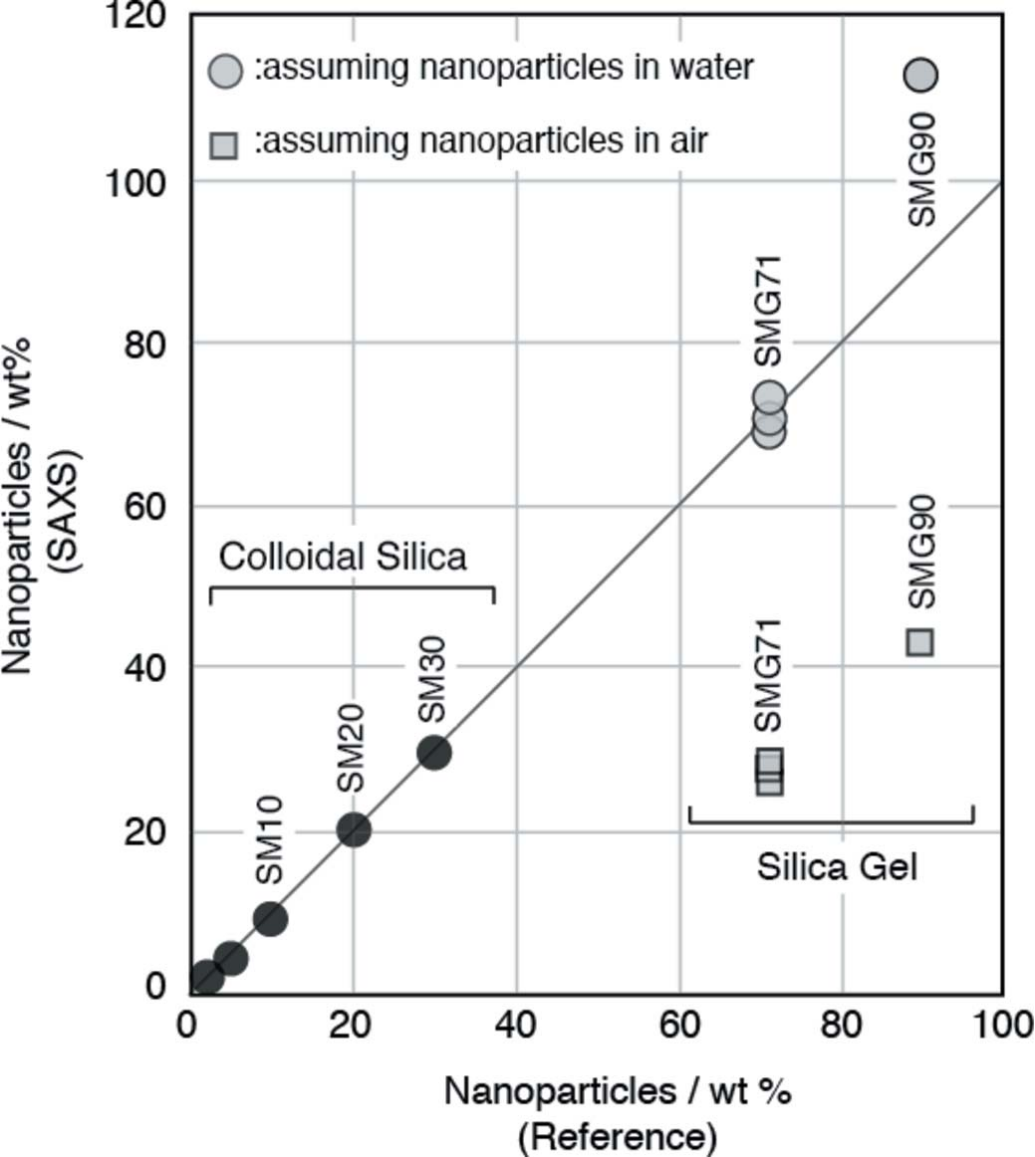
The weight ratios of nanoparticles in colloidal silica and silica gels determined with the integral SAXS method as a function of the reference weight ratios. The weight ratios of silica gels were calculated for two cases: nanoparticles in water and in air.

**Figure 5:**
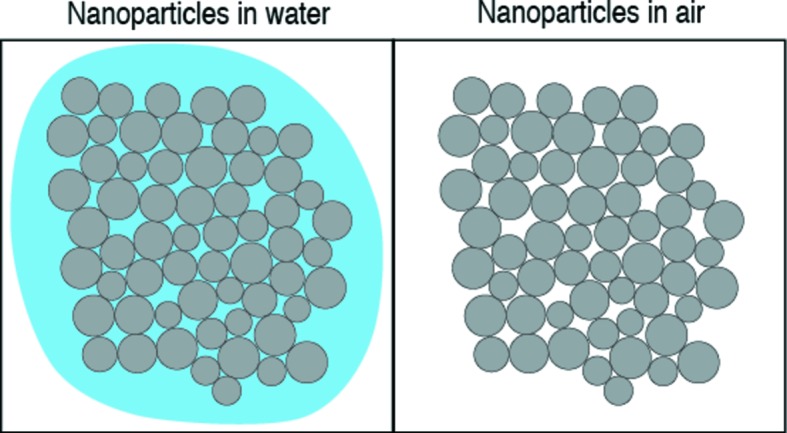
A schematic diagram showing nanoparticles in water (left) and in air (right).

**Figure 6:**
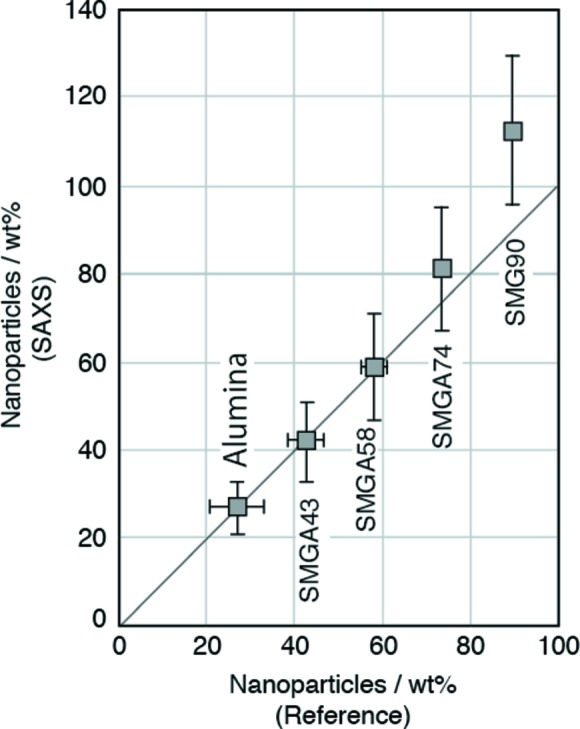
The weight ratios of nanoparticles in mixtures of α-alumina and silica gel. The reference weight ratio of nanoparticles in α-aluminium oxide was determined with the SAXS method on the assumption that the density of nanoparticles was the same as that of α-alumina.

**Figure 7:**
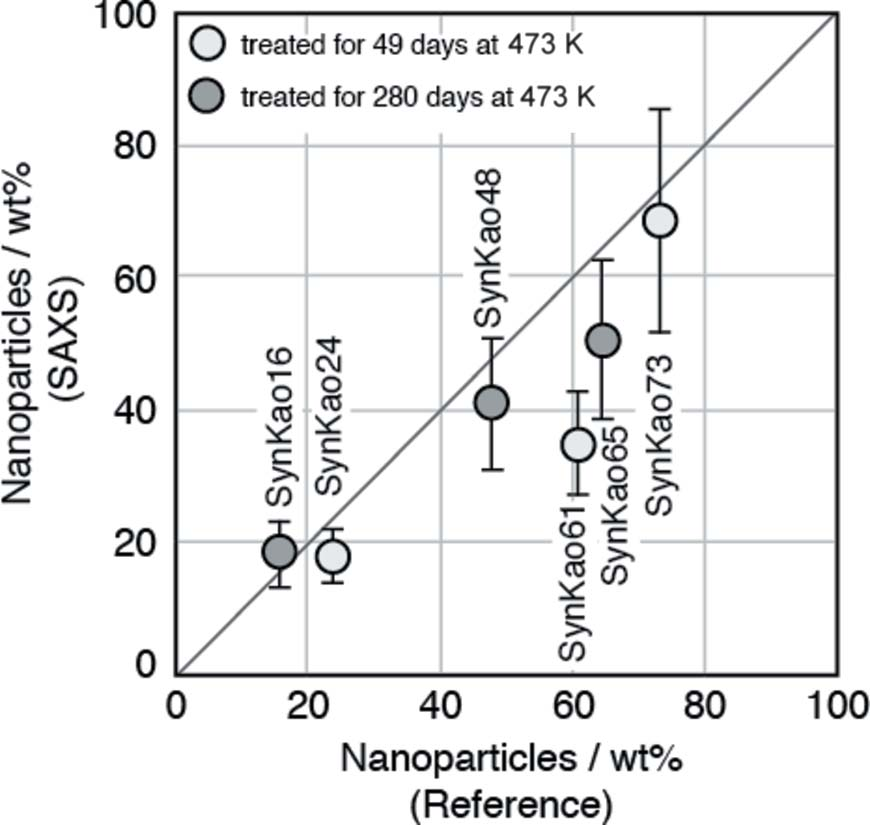
The weight ratio of nanoparticles in synthetic clays determined with the integral SAXS method. The reference weight ratios of nanoparticles were estimated from the weight ratio of kaolinite on the assumption that the samples contain only kaolinite and amorphous nanoparticles.

Table 5 ▸ shows the weight ratios of nanoparticles determined with the integral SAXS method and their reference weight ratios. Fig. 4 ▸ shows the weight ratios of nanoparticles measured with the integral SAXS method for the colloidal silica and silica gels as a function of the reference weight ratios. The weight ratios of the nanoparticles are very close to the reference weight ratios for colloidal silica and silica gel SMG71. We calculated the weight ratios of nanoparticles in the silica gels with two models: nanoparticles are present in water and in air (Fig. 5 ▸). The interstitials of nanoparticles in SMG71 will be completely filled with water, but the interstitials of nanoparticles in SMG90 will be partially filled with water. Fig. 6 ▸ shows the weight ratios of nanoparticles in the mixtures of α-alumina and SMG91 determined with the integral SAXS method as a function of the reference weight ratios. The sample of α-alumina contains a considerable proportion of nanoparticles. The weight ratios of the nanoparticles in the mixtures are close to the reference weight ratios, except for SMG90. Fig. 7 ▸ shows the weight ratios of nanoparticles in the synthetic clays determined with the integral SAXS method as a function of the reference weight ratios. The weight ratios of nanoparticles in the synthetic clays are also close to the reference weight ratios except for SynKao61.

### 4.2. Size distribution and aggregation state of nanoparticles   

Table 6 ▸ shows the average and the standard deviation of the external radius for the volume distribution of spherical nanoparticles. The average radii of colloidal silica and silica gels range from 4.2 to 5.4 nm. These radii are 20–54% larger than the value (3.5 nm) listed in the brochure of Grace Davison for SM30. Transmission electron microscopy (TEM) observation of SM30 also shows that the radii are somewhat larger than 3.5 nm. Borchert *et al.* (2005 ▸) confirmed that the average size of nanoparticles determined with a SAXS method is close to that observed with TEM. The synthetic clays have average radii from 2.9 to 4.6 nm, and the natural clays have average radii from 4.2 to 7.4 nm. The radii of voids range from 1.4 to 1.7 nm for the colloidal silica and silica gels, 1.1 to 1.5 nm for the synthetic clays and 1.6 to 1.7 nm for the natural clays.

**Figure 8:**
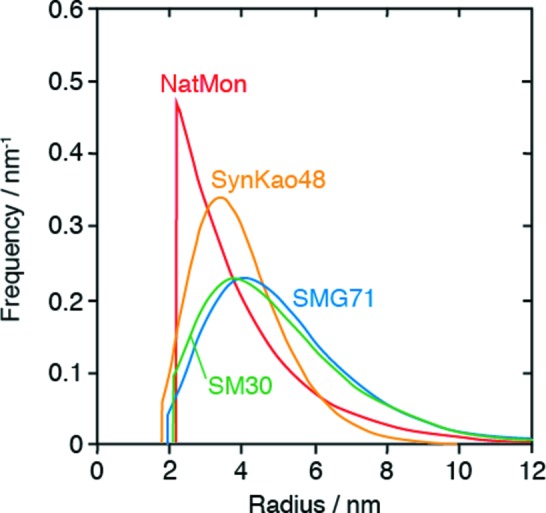
The volume distributions of nanoparticles as a function of radius for SM30, SMG71-2, SynKao48 and NatMon.

**Figure 9:**
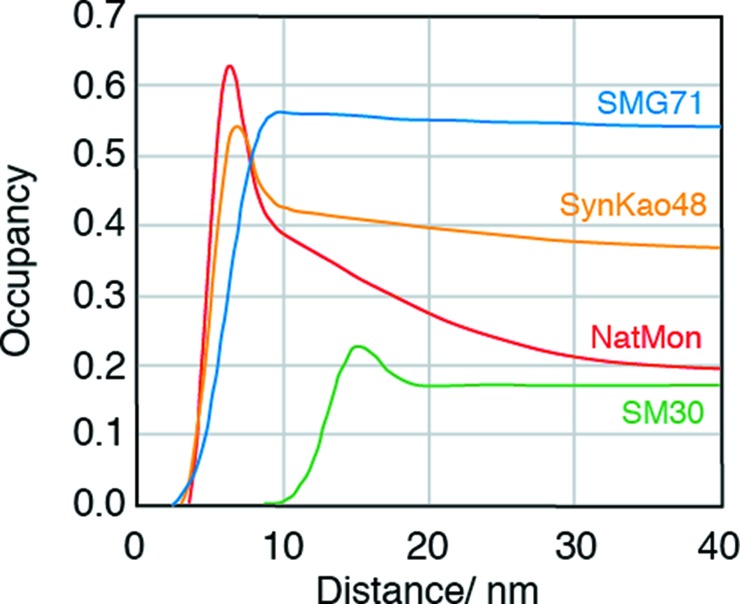
The Patterson functions of the nanoparticle centers for SM30, SM71G-2, SynKao48 and NatMon.

**Figure 10:**
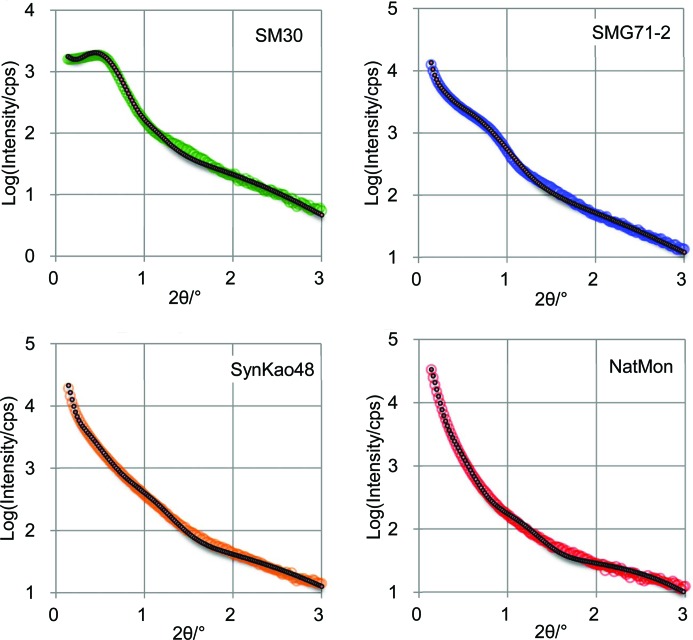
Observed (colored) and calculated (black) scattering intensities for SM30, SMG71-2, SynKao48 and NatMon.

**Figure 11:**
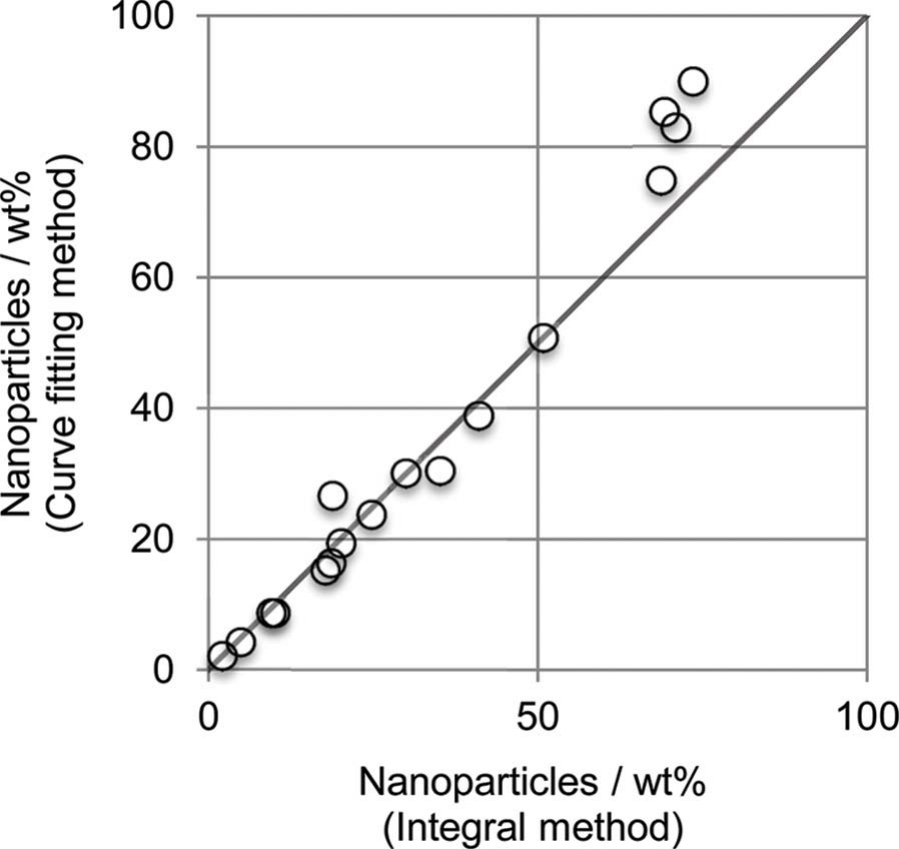
The weight percent of nanoparticles determined with the curve-fitting SAXS method as a function of those determined with the integral SAXS method.

Fig. 8 ▸ shows the volume frequencies of nanoparticles as a function of external radius, Fig. 9 ▸ shows the Patterson functions of the centers of the nanoparticles, and Fig. 10 ▸ shows the observed intensities and the calculated intensities for SM30, SMG71-2, SynKao48 and NatMon. These volume frequencies and Patterson functions were used for calculating SAXS intensities. In this calculation, we have obtained the scale factor (*K*_obs–cal_) between observed and calculated SAXS intensities. From the scale factor we can also calculate the weight ratio of nanoparticles. Fig. 11 ▸ shows the weight ratios calculated from the scale factor as a function of the weight ratio of nanoparticles calculated from the integral SAXS method. The weight ratios of nanoparticles calculated from the scale factor *K*_obs–cal_ are very close to those calculated with the integral SAXS method. This confirms that all the SAXS except for the background and the peak of the clay mineral is elastic scattering from nanoparticles.

## 5. Discussion   

### 5.1. Integral SAXS method   

We have developed a method to quantify nanoparticles in clays and soils from SAXS intensities. By integrating the SAXS intensities over three-dimensional reciprocal space we obtain a value that is proportional to the weight ratio of the nanoparticles. The method is simple and easy because one does not need to calculate SAXS intensities from the size distribution and aggregation state of nanoparticles. However, as mentioned above, we still have several problems for the integral SAXS method. These problems are discussed in the following sections.

### 5.2. Normalized intensities   

We have proposed a new method to derive normalized intensities, which are the intensities scattered by unit weight of a sample from unit intensity of the incident beam with no absorption effect. We have found that we can derive normalized intensities from the incident direct-beam intensity, the transmission factor and the chemical composition of the sample. The validity of the derivation has been confirmed by the results that the weight ratios of nanoparticles in colloidal silica and silica gel SMG71 are determined accurately. This new method enables us to calculate normalized intensities more easily than with a conventional method that uses an internal standard to derive normalized intensities (Alexander & Klug, 1948 ▸). To apply the conventional method to SAXS, we need standard crystalline materials that are pure and have a diffraction peak in the SAXS area, but such crystals are rare. Candidates for such crystals are phyllosilicate minerals, such as mica and talc. We tried to use these minerals as an internal standard, but we found that they were inadequate for this purpose. These minerals cannot be ground sufficiently and have strong preferred orientation because the crystals have the shape of a wide flat plate. This makes the peak intensity of the standard sample vary from sample to sample.

### 5.3. Densities of nanoparticles   

We need to know the density of the nanoparticles to determine their weight ratios from the integral SAXS intensity because the integral SAXS intensity is proportional to the weight ratio of nanoparticles, proportional to the square of the difference of density between the nanoparticles and the liquid surrounding the nanoparticles, and inversely proportional to the density of nanoparticles. However, measuring the density of nanoparticles in clays and soils is difficult. Therefore, we have calculated the density of nanoparticles from the chemical composition of a sample by assuming that the chemical composition of the nanoparticles is the same as that of the sample, and that the volume of the nanoparticles is proportional to the weight ratio of oxygen atoms. These assumptions result in large errors of about 12% of the density. This large error in density results in a large error in estimated weight ratio of the nanoparticles, which is about 19% of the weight ratio of the nanoparticles.

We can reduce the error of the weight ratio of the nanoparticles determined with the integral SAXS method by reducing the error of the density of the nanoparticles. We may be able to determine the densities of nanoparticles more accurately by obtaining two sets of intensity data measured under conditions where the densities of the materials surrounding the nanoparticles are different. For example, by obtaining the intensity data for nanoparticles in pure water and the intensity data for those in air, we may be able to determine the density of the nanoparticles more accurately.

We assume that the interstices of the nanoparticles are completely filled with water, but the interstices of the nanoparticles in some clays or soils may be partially filled with water. In fact, the interstices of the nanoparticles in SMG90 are partially filled with water. The error from this effect is about 14%. By placing the samples in high water vapor pressure conditions, we will ensure that the interstices of the nanoparticles are completely filled with water, which will reduce the error for quantifying the nanoparticles.

### 5.4. Scale factor between the integral intensity and the weight ratio of nanoparticles   

We need to determine the scale factor (*K*) between the integral intensity and the weight ratio of nanoparticles using equation (9). To determine the scale factor, we have measured the SAXS intensities of a standard sample that has a known quantity of nanoparticles. We used colloidal silica (SM30) as the standard sample because this colloidal silica contains a large, known quantity of nanoparticles and is commercially available. Once we determine the scale factor, we do not need to redetermine it unless we change the diffractometer or the optical system of the diffractometer.

### 5.5. Removing the diffraction intensities from a clay mineral   

When a diffraction peak of a clay mineral is present in the SAXS area, we need to remove the peak before integrating the SAXS intensities. We have fitted the peak with a normal distribution or two kinds of normal distribution so as to make the subtracted intensities decrease monotonically and smoothly. The selection of the peak intensity and shape is, however, somewhat arbitrary. This results in an error of about 10% of the weight ratio. It would be better to remove the peak from the clay mineral automatically by developing an algorithm to determine the shape of the peak uniquely.

### 5.6. Validity and errors of the integral SAXS method   

We have excluded scattering other than the elastic scattering of nanoparticles from the SAXS. By subtracting the background, which was determined from the average intensity around 9° 2θ, we have excluded the inelastic scattering (*e.g.* fluorescent X-rays from iron) and the scattering from air. By subtracting the diffraction peak of the clay mineral in the SAXS area, we can exclude the scattering from the clay mineral. Even if we subtract the background and the diffraction peak, we still have the question of whether or not SAXS without the background and without the diffraction peak includes scattering other than the elastic scattering from nanoparticles. For example, the SAXS might include scattering originating from stacking faults of a clay mineral. In this case, the weight ratio of nanoparticles determined with the integral method is overestimated. Therefore, we have determined the weight ratio of nanoparticles by fitting the observed intensities with the calculated intensities, where the calculated intensities were derived on the basis of the size distribution and aggregation state of the nanoparticles. In this calculation, we can obtain the scale factor between observed and calculated intensities (*K*_obs–cal_). The scale factor gives us the weight ratio of nanoparticles. These weight ratios were close to those determined with the integral SAXS method (Fig. 11 ▸). This shows that SAXS from the studied clays originated only from nanoparticles.

The integral SAXS method to quantify nanoparticles in clays and soils has several sources of error. The largest error comes from the error of the estimated density of nanoparticles, which is about 19% of the weight ratio of the nanoparticles. The second largest error comes from the assumption that the interstices of nanoparticles are completely filled with water. Some part of the interstices of nanoparticles might not be filled with water. This error is about 14% of the weight ratio of nanoparticles. The third largest error comes from the error during peak removal, which is about 10% of the weight ratio of nanoparticles. Additional errors amount to 5% of the weight ratio of nanoparticles. The total errors are about 25% of the weight ratio of nanoparticles.

### 5.7. Presence of voids in nanoparticles   

We find that the nanoparticles in colloidal silica and silica gels have spherical voids at the center. We reach this conclusion because the calculated intensities determined from the model with voids fit the observed intensities very well, but those determined from the model without voids do not. On the other hand, for synthetic and natural clays, SAXS cannot determine which models are valid. This is because the intensities calculated with the two models (with voids and without voids) both fit the observed intensities very well. However, the nanoparticles in allophane, which is the only aluminium silicate system so far reported, have voids at the center (Wada & Wada, 1977 ▸; Creton *et al.*, 2008 ▸; Iyoda *et al.*, 2012 ▸). Therefore, we assume that the nanoparticles in clays also have voids at the center when we calculate the SAXS intensities. Although the voids in the nanoparticles may not be exactly spherical, the shapes are similar to a sphere. Therefore, we have calculated SAXS intensities by assuming that the voids are spherical. However, we do not need to determine whether or not the nanoparticles have voids at their centers when we quantify nanoparticles with the integral SAXS method.

### 5.8. Nanoparticles in clays and soils   

Nanoparticles have been observed ubiquitously in volcanic soils with TEM (Kitagawa, 1971 ▸; Henmi & Wada, 1976 ▸; Karube *et al.*, 1996 ▸; Jongmans *et al.*, 2000 ▸; Woignier *et al.*, 2007 ▸, 2008 ▸; Matsuura *et al.*, 2013 ▸). Some amorphous aluminium silicates synthesized below 373 K also contain large quantities of nanoparticles (Suzuki *et al.*, 2009 ▸). Most silicate materials formed at low temperatures, such as clays and soils, seem to contain a large fraction of nanoparticles. However, the nanoparticles in clays and soils have not been quantified to date because there was no established method to do so. In this study, we have developed a method to quantify nanoparticles in clays and soils and have quantified the nanoparticles in three clays. We have found that these clays contain large quantities (10–25 wt%) of nanoparticles. We expect that clays and soils other than volcanic soils will also contain a considerable number of nanoparticles because such clays and soils have strong SAXS intensities. We are preparing to measure the weight ratios of nanoparticles in clays and soils from many localities and to compare the weight ratios of the nanoparticles with the properties of the clays and soils.

## 6. Conclusion   

We have demonstrated a method to quantify the nanoparticles in clays and soils with an integral SAXS method. Normalized intensities were derived from raw intensity data. By integrating the normalized intensities over three-dimensional reciprocal space, we have obtained a value proportional to the weight ratio of the nanoparticles and to the difference of density between the nanoparticles and the liquid surrounding the nanoparticles, and inversely proportional to the density of the nanoparticles. The density of nanoparticles in clays was estimated from the chemical composition of the sample. Then, we calculated the weight ratio of nanoparticles from the integrated intensity and the density of nanoparticles. This weight ratio of nanoparticles determined with the integral SAXS method has an error of about 25% of the weight ratio of the nanoparticles. The weight ratios of nanoparticles in three kinds of natural clays measured in this study range from 10 to 25 wt%.

## Figures and Tables

**Figure 12:**
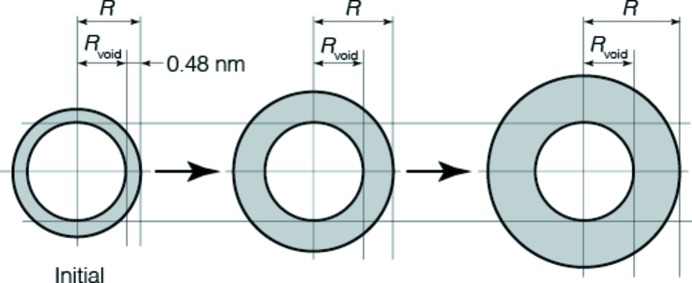
A growth model of nanoparticles in clays and soils. Initially, nanoparticles consist of a spherical shell of 0.48 nm thickness. Then, the shell grows outwards and becomes thicker with time. *R*_void_ is constant, but *R* increases with time.

**Figure 13:**
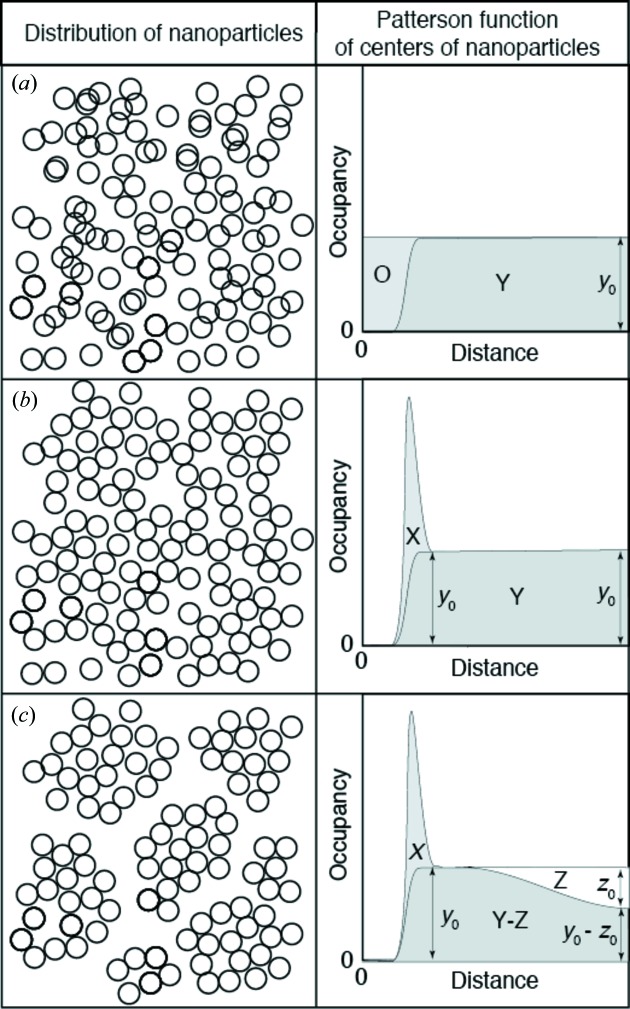
Relation between the distribution of centers of nanoparticles and their Patterson functions. ‘Occupancy’ on the vertical axis means the average volume ratio occupied by nanoparticles at the position whose distance from a reference nanoparticle is ‘Distance’. (*a*) When the nanoparticles are distributed completely randomly, the Patterson function is constant. The area labeled ‘O’ corresponds to overlapping spheres. (*b*) When the nanoparticles are distributed randomly but do not overlap each other, the area labeled ‘O’ in (*a*) moves to the area ‘X’. The area ‘X’ corresponds to spheres in contact with each other. (*c*) When the nanoparticles form secondary particles, the Patterson function decreases with increasing distance.

**Table 1:** List of symbols

Symbol	Meaning
*a*	Cross section of the sample that scatters the X-ray beam
*c_i_*	Number ratio of the *i*th nanoparticle (= *N_i_*/*N*)
d*c*	Number ratio of nanoparticles whose radius is in the range between *R* and *R* + d*R*
d*v*	Volume ratio of nanoparticles whose radius is in the range between *R* and *R* + d*R*
Δ*R*	Increment of *R* (radius of the nanoparticle) for the calculation of *F_i_*(**q**)
Δ*r*	Increment of |**r**| for the calculation of *s*(**r**)
Δρ	Difference of density between nanoparticles and the liquid surrounding the nanoparticles
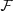	Fourier transform
*f_i_*(**r**)	Density of *i*th kind of nanoparticle when the center is at the origin
*F_i_*(**q**)	Form factor for *i*th kind of nanoparticle
*g_p_*(**r**)	Density of the *p*th nanoparticle
*I*(**q**)	Scattering intensity
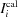	Calculated scattering intensity at the *i*th position
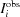	Observed scattering intensity at the *i*th position
*I*_0_	Intensity of direct incident beam
*I*_raw_	Raw intensity of scattered beam
*I*_norm_	Normalized intensity of scattered beam
*I*_trans_	Intensity of direct transmitted beam
*K*	Scale factor between the integral SAXS intensity and the value of (Δρ)^2^(*W*/ρ_nano_)
*K*_obs–cal_	Scale factor between observed and calculated intensities
*L*	Number of kinds of spheres of different size
*l*	Sequential number of nanoparticle with radius of *l*Δ*R*
*M*	Mass of the sample that scatters the X-ray beam
μ	Absorption coefficient of sample
*m*_log(*v*)_	Average for a volume logarithmic normal distribution
(μ/ρ)_*k*_	Mass absorption coefficient of element *k*
*N*	Total number of nanoparticles ( 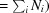
*N_i_*	Number of the *i*th kind of nanoparticles
*P*(**r**)	Patterson function of nanoparticles
**q**	Position in reciprocal space
**r**	Position in real space
*R*	Radius of nanoparticle
*R*_factor_	Residual factor
*R_i_*	Radius of *i*th nanoparticle
*R*_void_	Radius of voids in nanoparticles
ρ	Density of the sample that scatters the X-ray beam
ρ_nano_	Density of nanoparticles
*s*(**r**)	Probability that we find the center of a nanoparticle at **r** + **r**_0_ when the center of another nanoparticle is present at **r**_0_
*S*(**q**)	Structure factor
*s_ij_*(**r**)	Probability that we find the *j*th kind of nanoparticle at **r** + **r**_0_ when the center of an *i*th kind of nanoparticle is present at **r**_0_
σ_log(v)_	Standard deviation for a volume logarithmic normal distribution
*t*	Length of the sample that scatters the X-ray beam
*u_l_*	Value of *s*(**r**) when |**r**| = *l*Δ*r*
*V*	Volume of the sample that scatters the X-ray beam
*V*_ext_	External volume of nanoparticles including the central void part
*V_p_*	Volume of the *p*th nanoparticle
*V*_total_	Total volume of nanoparticles in a unit weight of sample
*V*_true_	Volume of nanoparticles excluding the central void part
*W*	Weight ratio of nanoparticles in a sample
*w_k_*	Weight ratio of element *k* in a sample
*x_l_*	Concentration of surrounding nanoparticles that are in contact with a reference nanoparticle when *l* = |**r**|/Δ*r*
*y_l_*	Concentration of surrounding nanoparticles that are not in contact with a reference nanoparticle and are inside of the secondary particle containing the reference nanoparticle when *l* = |**r**|/Δ*r*
*y_l_* − *z_l_*	Concentration of surrounding nanoparticles that are not in contact with a reference nanoparticle and are outside of the secondary particle containing the reference nanoparticle when *l* = |**r**|/Δ*r*

**Table 2:** Sample descriptions

Kind of sample	Sample name	Preparation method of samples
Colloidal silica	SM30	Purchased from Grace Davison
	SM20	Prepared by diluting SM30 with water
	SM10	
	SM5	
	SM2	

Silica gel	SMG71	Prepared by evaporating water from SM30
	SMG90	

Aluminium oxide	Alumina	α-Aluminium oxides purchased from Alfa Aesar

Mixtures of silica gel and α-alumina	SMGA74	SMG90 mixed with α-alumina
	SMGA58	
	SMGA43	

Synthetic clay	SynKao73	Hydro­thermally synthesized from aluminium silicate gel at 473 K
	SynKao65	
	SynKao61	
	SynKao48	
	SynKao24	
	SynKao16	
		
Natural clay	NatKao	Kaolinite clay from Kanpaku deposit†
	NatPyr	Pyrophillite clay from Shokozan deposit†
	NatMon	Montmorillonite clay from Mikawa deposit†

† A reference sample of the Clay Science Society of Japan.

**Table 3:** Chemical compositions and status of samples for colloidal silica, silica gels, mixtures of silica gel and α-aluminium oxide, and synthetic clays

	Chemical composition (wt%)			
Sample name	SiO_2_	Al_2_O_3_	H_2_O	Status
SM30	30.0	0.0	70.0	Wet
SM20	20.0	0.0	80.0	Wet
SM10	10.0	0.0	90.0	Wet
SM5	5.0	0.0	95.0	Wet
SM2	2.0	0.0	98.0	Wet
SMG71-1	71.2	0.0	28.8	Dry
SMG71-2	71.2	0.0	28.8	Dry
SMG71-3	71.2	0.0	28.8	Dry
SMG90	89.7	0.0	10.3	Dry
Alumina	0.0	100.0	0.0	Dry
SMGA74	67.3	25.0	7.7	Dry
SMGA58	44.9	50.0	5.1	Dry
SMGA43	22.4	75.0	2.6	Dry
SynKao73	64.8	23.5	11.7	Dry
SynKao65	65.4	23.8	10.8	Dry
SynKao61	55.2	31.2	13.6	Dry
SynKao48	55.6	31.5	12.9	Dry
SynKao24	46.5	39.4	14.1	Dry
SynKao16	46.2	39.2	14.6	Dry

**Table 4:** Chemical compositions of samples for natural clays

	Chemical composition (wt%)										
Sample name	SiO_2_	TiO_2_	Al_2_O_3_	Fe_2_O_3_	MgO	CaO	Na_2_O	K_2_O	P_2_O_5_	H_2_O	Status
NatKao	43.9	0.1	36.6	0.0	0.0	0.0	0.1	0.8	0.2	18.3	Dry
NatPyr	67.9	0.2	23.3	0.2	0.0	0.0	0.1	0.1	0.0	8.5	Dry
NatMon	66.4	0.1	11.9	1.6	2.6	0.5	2.0	1.3	0.0	13.6	Dry

**Table 5:** Weight ratios of nanoparticles determined with the integral SAXS method and their reference values Estimated errors are shown in parentheses.

	Nanoparticle (wt%)		
Sample name	Determined with SAXS	Reference	Comments on reference values
SM30	30.0	30.0	Taken from brochure of LUDOX

SM20	20 (1)	20.0	Estimated from the amount of water added to SM30
SM10	9.5 (5)	10.0	
SM5	4.6 (3)	5.0	
SM2	2.1 (1)	2.0	

SMG71-1	74 (4)	71.2	Estimated from the amount of water evaporated from SM30
SMG71-2	69 (4)	71.2	
SMG71-3	71 (4)	71.2	
SMG90	112 (17)	89.7	

Alumina	27 (6)	27 (6)	Estimated with the integral SAXS method

SMGA74	82 (14)	74 (2)	Estimated from the mixing ratio between SMG90 and α-alumina
SMGA58	59 (12)	58 (3)	
SMGA43	42 (9)	43 (4)	

SynKao73	69 (17)	73 (2)	Estimated from kaolinite weight ratio on the assumption that samples consist of only kaolinite and amorphous nanoparticles
SynKao65	51 (12)	65 (2)	
SynKao61	35 (8)	61 (2)	
SynKao48	41 (10)	48 (1)	
SynKao24	18 (4)	24 (1)	
SynKao16	19 (5)	16 (1)	

NatMon	25 (7)		
NatPyr	10 (2)		
NatKao	19 (5)		

**Table 6:** Average and standard deviation of external radius of nanoparticles, and radius of void in nanoparticles Estimated errors are shown in parentheses.

	Distribution of external radius		
Sample name	Average (nm)	Standard deviation (nm)	Radius of void (nm)
SM30	4.97 (4)	2.00 (8)	1.60 (2)
SM20	4.85 (4)	1.98 (7)	1.60 (2)
SM10	4.65 (4)	1.74 (8)	1.57 (3)
SM5	4.48 (8)	1.82 (9)	1.66 (3)
SM2	4.2 (2)	1.7 (2)	1.74 (5)
SMG71-1	5.11 (4)	1.90 (6)	1.40 (2)
SMG71-2	5.19 (4)	2.06 (7)	1.45 (2)
SMG71-3	5.03 (4)	1.92 (7)	1.44 (3)
SMG90	5.38 (3)	1.97 (5)	1.45 (2)
Alumina	9.3 (2)	5.0 (1)	1.62 (3)
SynKao73	2.9 (2)	1.2 (3)	1.12 (3)
SynKao65	4.36 (4)	1.63 (9)	1.45 (3)
SynKao61	3.79 (4)	1.22 (8)	1.35 (3)
SynKao48	4.01 (3)	1.31 (5)	1.33 (2)
SynKao24	4.60 (5)	1.51 (9)	1.38 (2)
SynKao16	4.07 (4)	0.9 (1)	1.45 (4)
NatMon	4.2 (3)	0.9 (4)	1.70 (2)
NatPyr	6.5 (2)	3.8 (5)	1.73 (5)
NatKao	7.4 (4)	3.8 (2)	1.63 (4)

## Appendix A

We here derive the relation between *P*(**0**) and *W* (the weight ratio of nanoparticles). We consider the nanoparticles in a unit weight of sample. We express the difference of density between the *p*th nanoparticle and the liquid surrounding the naonparticle with a function . When  is in the *p*th nanoparticle, , and when  is outside of the *p*th nanoparticle, , where  is the difference of density between the nanoparticle and the liquid surrounding the nanoparticles. The sum of the differences of density for all of the nanoparticles is expressed as

The Paterson function of  is expressed as

When ,

Because two nanoparticles do not overlap each other,  when . Therefore,

where *V_p_* is the volume of the *p*th nanoparticle, *V*_total_ is the sum of the volumes of all the nanoparticles, *W* is the weight ratio of nanoparticles in a sample and ρ_nano_ is the density of nanoparticles.

## Appendix B

We here derive the form factors [*F_i_*(**q**)]. We consider a spherical nanoparticle that has a spherical external shape and a spherical void at the center:

where *R*_void_ and *R_i_* are the radius of the void and the external radius of the *i*th kind of spherical nanoparticle, respectively. When *R*_void_ = 0, *f_i_*(**r**) is a sphere without a void. We have proposed this shape of nanoparticle from the growth model shown in Fig. 12 ▸. We consider that the initial shape of the nanoparticle is a spherical shell and that the thickness of the wall is 0.48 nm. The external radius increases with time, but *R*_void_ is constant. Form factors *F_i_*(**q**) can be derived from the Fourier transform of equation (20):

where *q* = |**q**|.

## Appendix C

We here derive the number ratio of the *i*th nanoparticle (*c_i_*). We assume that the size distribution of spherical nanoparticles is expressed with a volume logarithmic normal distribution. In such a case, the summation of the volume of spherical nanoparticles whose logarithm of radius is in the range between log(*R*) and log(*R*) + d[log(*R*)] is expressed as

where *m*_log(*v*)_ and σ_log(*v*)_ are the average and the standard deviation on a logarithmic scale. Because d[log(*R*)] = d*R*/[*R*ln(10)], the volume of spheres whose radius is in the range between *R* and *R* + d*R* is expressed as

The number ratio of spheres whose radius is in the range between *R* and *R* + d*R* is expressed as

Next, let us approximate the number ratio of spheres with a discrete function because we calculate the intensities with a discrete function as in equation (14). If *L* kinds of sphere with a radius of *R_i_* (= *i*Δ*R*, *i* = 1 to *L*) are present, then the ratio of the spheres with radius *R_i_* is expressed as

Using the values of *R_i_* and *c_i_*, the average of the external volume of the nanoparticles, *V*_ext_, is calculated with the following equation:

The average of the volume of nanoparticles excluding the volume of the central void, *V*_true_, is calculated with the following equation:

## Appendix D

We here derive the structure factor [*S*(**q**)]. First, we consider the shape of the Patterson function of the nanoparticle centers [*s*(***r***)]. Fig. 13 ▸(*a*) shows the nanoparticles distributed randomly; the random distribution means that some nanoparticles may overlap each other. In that case, the Patterson function is constant [Fig. 13 ▸(*a*)]. Let us move these nanoparticles so that they do not overlap each other. With this operation, the nanoparticle pairs that overlapped each other will be in contact with each other, which moves the area labeled ‘O’ in the Patterson function in Fig. 13 ▸(*a*) to the area labeled ‘X’ in Fig. 13 ▸(*b*). The area labeled ‘Y’ in Fig. 13 ▸(*b*) corresponds to the correlation function proposed by Debye (1927 ▸), and the area labeled ‘X’ is missing in the model of Debye (1927 ▸). By adding the area labeled ‘X’, we can avoid the problem that the calculated intensities close to |**q**| = 0 become negative when the occupancy rate of spheres exceeds one-eighth. We also consider the case where secondary particles are present [Fig. 13 ▸(*c*)]. In this case, the Patterson function has a large value when the distance is small, but decreases with increasing distance and approaches the average value of the whole space. The area labeled ‘Z’ in Fig. 11 ▸(*c*) is zero for colloidal nanoparticles but will have positive values for nanoparticles in inhomogeneous media such as clays and soils.

Next, we show how we approximate the Patterson function of nanoparticle centers with a step function:

where

The values of *s_l_* are expressed with the summation of three terms:

The first term, *x_l_*, is a normal distribution with the peak at *m_x_* and a standard deviation of σ_*x*_. This term corresponds to the area labeled ‘X’ in Fig. 13 ▸. The second term, *y_l_*, increases with increasing *l*, having a value of *y*_0_/2 at *m_y_* and approaching *y*_0_ with increasing *l*. This term corresponds to the area labeled ‘Y’ in Fig. 13 ▸. The third term, *z_l_*, increases with increasing *l*, having a value of *z*_0_/2 at *m_z_* and approaching *z*_0_ with increasing *l*. This term corresponds to the area labeled ‘Z’ in Fig. 13 ▸.

The structure factor is derived from the Fourier transform of the Patterson function of the center positions of spherical nanoparticles. Because  when |**q**| > 0, the Fourier transform of the Patterson function of the nanoparticle centers is expressed as

We can finish the summation of  in equation (31) at a finite *l* value when the value of [*s_l_* − (*y*_0_ − *z*_0_)] is close to zero because the value of [*s_l_* − (*y*_0_ − *z*_0_)] approaches zero with increasing *l*. The Fourier transform of each spherical shell is approximated as
